# Fedbatchdesigner: A User-Friendly Dashboard for Modeling and Optimizing Growth-Arrested Fed-Batch Processes

**DOI:** 10.1021/acssynbio.5c00357

**Published:** 2025-07-21

**Authors:** Andrea C. Graf, Julian Libiseller-Egger, Mathias Gotsmy, Jürgen Zanghellini

**Affiliations:** † Department of Analytical Chemistry, University of Vienna, Vienna 1090, Austria, EU; ‡ Austrian Centre of Industrial Biotechnology GmbH, Vienna 1190, Austria, EU; § Doctoral School in Chemistry, University of Vienna, Vienna 1090, Austria, EU; ∥ Department of Chemistry and Applied Biosciences, ETH Zurich, Zürich 8093, Switzerland

**Keywords:** microbial fermentation, bioprocess design, growth-decoupled production, TRY optimization, web application

## Abstract

Optimizing fed-batch fermentation strategies is key to maximizing bioprocess efficiency. While mathematical modeling can aid process design, its complexity often limits accessibility for experimental scientists. We present FedBatchDesigner, a user-friendly web tool for optimizing fed-batch processes with a growth-arrested production stage. With minimal input requirements, FedBatchDesigner enables rapid exploration of a process’s titer, rate, and yield (TRY) landscape for constant, linear, and exponential feeding strategies. Interactive visualizations allow users to assess trade-offs between productivity and titer, supporting rational decision-making without the need for extensive modeling expertise. We demonstrate FedBatchDesigner’s utility via two case studies: synthesis of (i) l-valine with a microaerobic production stage in and (ii) ethanol under nitrogen starvation in . FedBatchDesigner is freely available at https://chemnettools.anc.univie.ac.at/FedBatchDesigner, with the source code provided at https://github.com/julibeg/FedBatchDesigner under the MIT license.

## Graphical abstract

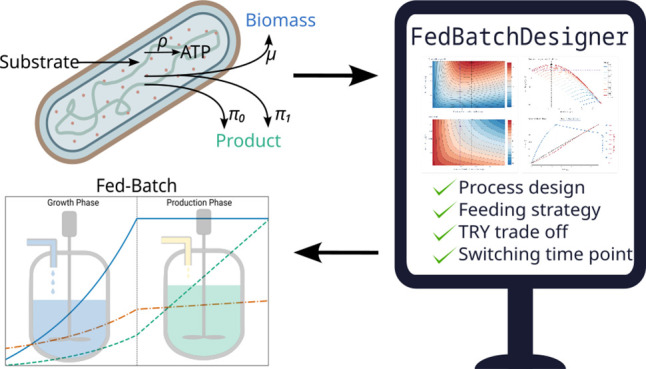

Biotechnology utilizes (micro)­organisms in the large-scale production of bulk chemicals or macromolecules for industrial or medical applications.^1^ Generally, the product of interest is synthesized at higher rates when more cells (biomass) are present. However, increasing biomass consumes substrate, which is then no longer available for product formation. Therefore, when designing a process, one usually faces the inherent tension between the TRY metrics: **Titer** (final product concentration), **Rate** (or average volumetric productivity; final product concentration divided by total process time), and **Yield** (total amount of product divided by the total amount of substrate used during the process).^2^

Fed-batch processes with a growth-arrested production stage [subsequently referred to as two-stage fed-batch (2SFB)] are an increasingly popular approach for achieving high process performance.^3–9^ They usually consist of the following stages:

(1) **Stage 0, initial batch phase.** After inoculation, the cells grow at their maximum rate until the substrate in the batch medium is depleted.
(2) **Stage 1, growth phase.** Feed medium, typically with high substrate concentrations, is continuously added at a controlled rate to increase biomass while preventing substrate accumulation (which could cause overflow metabolism, growth inhibition, or osmotic stress) and ensuring the process stays within the system’s oxygen transfer and cooling limits, which is particularly crucial at industrial scale.
(3) **Stage 2, production phase.** Conditions are adjusted to inhibit growth and maximize product formation from the consumed substrate. The feed rate is often decreased to reflect the reduced substrate uptake rate (as no substrate is needed for growth).

Examples of this basic pattern appear in all areas of modern bioprocessing. Growth in the production phase can be stopped in several ways, including genetically induced growth inhibition,^7^ transition from aerobic to microaerobic conditions,^6,10^ pH changes,^4^ or depletion of medium components essential for growth but not production.^8,11^ The key advantage of 2SFB processes is that, due to the separation of growth from production, they allow for better control over the trade-off between product titer and productivity. This control is achieved by adjusting the timing of the transition from the growth to the production stage. A prolonged growth phase generally enhances productivity, while an extended production stage results in higher yields.

Mathematical process models are convenient and cost-effective tools for optimizing the point of switching from growth to production (among other parameters, like temperature or aeration^12^) and they enable iterative improvements before committing to costly experimental validation.^8^ However, while very useful for researchers with a computational background, the available modeling frameworks can be complex and usually require programming skills for implementation and interpretation (e.g., ref 13) making them challenging for many experimental scientists to adapt and use.

We address this gap by providing a simple and user-friendly web tool called FedBatchDesigner. Its graphical interface is designed to quickly identify and optimize 2SFB feeding strategies based on a simple mechanistic characterization of cellular production capabilities. It is freely available for immediate use at https://chemnettools.anc.univie.ac.at/FedBatchDesigner. No registration is required, and no user data is stored permanently. The source code is provided under the MIT license at https://github.com/julibeg/FedBatchDesigner and at https://doi.org/10.5281/zenodo.15863161, allowing free use, modification, and distribution. A video demonstration is available at https://youtu.be/MXR3mR-Ayig and at https://doi.org/10.5281/zenodo.16080319.

## Features

FedBatchDesigner enables the design of optimal exponential, linear, or constant feeding strategies for a 2SFB. Users input basic process and reactor parameters and physiological characteristics of the production host (see Figure 1a). FedBatchDesigner then exhaustively evaluates the TRY metrics on a grid of values for the feed rate and switching time between the growth and production stages to identify the optimal strategy. To ensure fair comparisons, the total amount of substrate spent and the final reactor volume are kept the same for all simulations. As a result, the per-substrate yield becomes a linear function of the final titer (and thus the strategy with the greatest titer also provides the highest yield).

**Figure 1:**
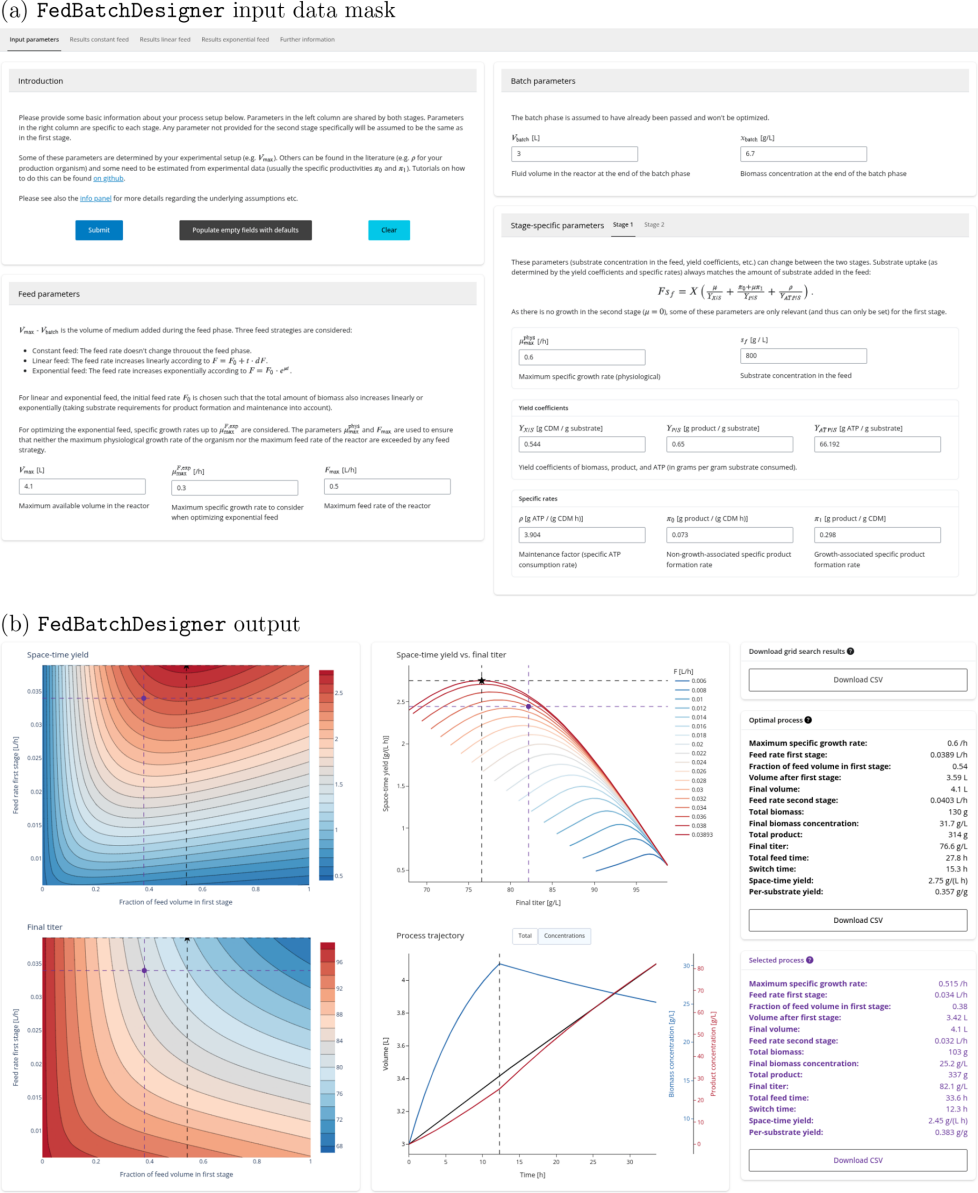
Screenshots of FedBatchDesigner’s input (a) and output (b) panels. Users can click on the interactive plots to select a process (purple circle) which is then compared with the optimal process (black star). Key metrics for both processes are summarized in the tables on the right. The left plots show space-time yield (top) and final titer (bottom) across combinations of feed rate and feed volume fraction in the first stage. The top right plot illustrates the trade-off between space-time yield and final titer, with each line representing a constant feed rate, linear growth rate, or exponential growth rate (depending on the result tab). The bottom right plot shows the time traces for biomass, product, and volume for the selected process.

Figure 1b shows FedBatchDesigner’s main outputs: several interactive plots that illustrate the trade-off between productivity and titer as functions of the feeding rate and the fraction of total feed volume spent in the growth phase. This fraction is a more natural choice for these visualizations than the time point of switching to the production phase as it allows for comparisons of processes with different feed rates in the growth phase which can have drastically different total process durations.

The plots highlight the optimal process (in terms of average volumetric productivity), but users can also click to select a nonoptimal process. This can be useful, for example, when choosing a process that is near-optimal, but has a lower feed rate during the growth phase, which may improve stability. The evolution of biomass, product, and reactor volume over time of the selected process is shown in the plot on the bottom right.

The results of the grid search can be downloaded in CSV format. The two tables on the right-hand side list characteristics of both the optimal and selected processes, which can also be downloaded as CSV. Plots can be exported as PNG images. Downloading the plots or CSV files facilitates easier comparison of different values for one or more input parameters: Users can run the analysis with their initial parameter set, save the plots and tables they are interested in, modify the value for one parameter, and run the analysis again to update the results. Users with programming experience can also clone and use the source code to explore the space of input parameters systematically with little adaptation.

## Implementation

FedBatchDesigner is implemented using Shiny for Python v1.3.0 ^14^ and Plotly v6.0.1 ^15^ for interactive visualizations. The source code is available at https://github.com/julibeg/FedBatchDesigner.

The main goal of FedBatchDesigner is to provide useful results while relying on few input parameters which can be easily estimated from limited amounts of experimental data. Additionally, the results should be easily interpretable and unlock intuitive insights. Therefore, the mathematical framework underlying our process model is based on the following simplifying assumptions, some of which are commonly used in fed-batch modeling^16,17^ whereas others are required to describe a 2SFB process:

(1) The process is performed in a spatially homogeneous, continuously stirred tank reactor and limited by a single substrate.
(2) The concentration of the limiting substrate is approximately zero throughout both feed phases as it is fully consumed by the cellular processes of maintenance, product formation, and growth.
(3) Host characteristics (yields, productivity coefficients, and cellular maintenance requirement) may vary between the two feed stages but remain constant within each stage.
(4) Growth is determined by the remaining substrate after accounting for maintenance and product formation.
(5) The specific product formation rate consists of a growth-associated and a nongrowth-associated term.
(6) Growth is negligible during the growth-arrested production stage.

These assumptions, in addition to requiring only very few parameters to be estimated from experimental data (for details see case studies below), also enable the derivation of analytical expressions for the evolution of biomass and product over time for all three feed types (see Section S1). Solving those is considerably faster than numerically integrating the underlying ordinary differential equations, which allows for exhaustively evaluating the space of possible feed strategies in little time.

In order to run an analysis, users must provide basic information about their process, limits on feasible feed rates, and stage-specific physiological data of the host organism (Figure 1a). Some inputs are determined by the fermentation setup (e.g., reactor volume), others can be found in the literature [e.g., adenosine triphosphate (ATP) yield on glucose for ],^18^ while the specific product formation rates will generally need to be fitted from prior data. Default physiological parameters for and , as well as all necessary values for the case studies discussed below (valine production with and ethanol production with ), can be preselected and loaded automatically.

After the user has entered the relevant parameters and the analysis has completed, space-time yield (i.e., average volumetric productivity) and final titer are visualized as functions of the feed rate as well as the relative amount of feed medium used during the growth stage (compared to the total feed volume; see previous section for details). Since the initial batch phase is not part of the 2SFB design, the TRY metrics displayed are based solely on the feed phase. Further, volume changes due to evaporation, sampling, and the addition of base solution for pH control are ignored.

FedBatchDesigner currently supports exponential, linear, and constant feeding strategies. However, our framework is designed for flexibility, allowing future extension to multiple stages and alternative feeding strategies. Process stages are represented as Python classes that inherit from an abstract base class and provide methods for computing biomass, product, and total volume at a given time point or after a certain amount of feed volume has been added. Additionally, while the current feed strategies are solved analytically, we provide a specialized base class for numerical integration of new and more sophisticated feeding strategies or process models. This requires only a method for evaluating the feed rate as a function of time and handles the remaining functionality automatically.

### Case Study 1: l-Valine Production

Hao et al.^6^ engineered for high-titer valine production, which they confirmed with two lab-scale fermentations. A standard fed-batch process reached a product titer of 86 g L^–1^, a yield of 0.33 g g^–1^ glucose, and an average volumetric productivity of 1.95 g L^–1^ h^–1^. In contrast, a 2SFB with a microaerobic production stage achieved a similar titer (84 g L^–1^) but with substantially improved yield (0.41 g g^–1^) and productivity (2.33 g L^–1^ h^–1^).

Using their data, we estimated the growth-associated and nongrowth-associated specific productivities (see the Jupyter Notebook at ref 19 for details) and then plugged these values into FedBatchDesigner (Figure 1a) to visualize the productivity–titer trade-off (Figure 1b). Before discussing the results below, it should be noted that Hao et al.^6^ dynamically controlled the feed rate during their fermentations, while we consider strictly constant, linear, or exponential feed strategies in our framework.

We find that considerably higher average productivities may be achieved by increasing the feed rate during the growth stage. However, Hao et al.^6^ already used a relatively high feed rate (corresponding to specific growth rates of up to 0.52 h^–1^). Thus, further increases might run the risk of exceeding the maximum growth rate early in the fed-batch which would lead to substrate accumulation (for comparison, the specific growth rate of W3110 on glucose M9 medium is 0.59 h^–1^).^20^ Further, the transition from growth to the production phase was almost optimally timed by Hao et al.^6^ and extending the growth phase from 12 h to 17 h would improve productivity by only 5% (cf. the optimal process with the selected process in Figure 1b).

However, employing a linear or exponential (rather than constant) feed strategy during the growth stage has the potential to further improve average volumetric productivity. For example, with a specific growth rate of μ = 0.3 h^–1^ and a batch duration of 4 h, a 2SFB with exponential feed can achieve a productivity of 2.79 g L^–1^ h^–1^ at a slightly reduced titer of 78 g L^–1^ (see Figure 2). Compared to the 2.33 g L^–1^ h^–1^ and 84 g L^–1^ reported by Hao et al.,^6^ this is an improvement of 20% in productivity with only a 7% reduction in titer.

**Figure 2:**
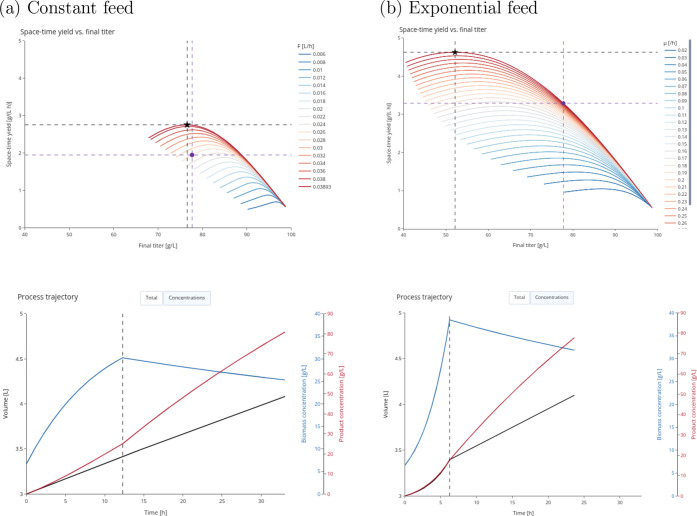
Comparison of l-valine production using a 2SFB with a microaerobic production phase and constant feed (a) or exponential feed (b) in the growth phase. The selected process (purple circle) in (a) corresponds to the feed strategy used by Hao et al.^6^ Using similar constant feed rates, only moderate improvements in space-time yield (i.e., average volumetric productivity) can be achieved. With an exponential feed strategy (b), however, larger gains in space-time yield are possible.

### Case Study 2: Enhanced Ethanol Production with ATP Wasting

Zahoor et al.^21^ created an strain with enforced ATP wasting that displayed improved substrate uptake and ethanol formation rates under nitrogen starvation, making it better suited to produce ethanol in a 2SFB setting with high volumetric productivity.

While Zahoor et al.^21^ did not perform bioreactor cultivations in their study, FedBatchDesigner can be used to inform the setup of the first 2SFB experiments in follow-up research. To illustrate how this can be done, we used their shake flask data to estimate the parameters required by FedBatchDesigner (see the notebook at ref 22 for details) and entered them into the interface (alongside reasonable values for a lab-scale bioreactor and physiological parameters for taken from the literature). Surprisingly, the results revealed that, despite the enforced ATP wasting, a one-stage fed-batch actually still achieves higher volumetric productivities than having a separate growth-decoupled production stage with nitrogen starvation. Indeed, with the chosen set of parameters, the growth-decoupled ethanol formation rate in the production phase would need to increase by at least a factor of 3 for a two-stage fed-batch to make sense in this case (see Figure S2). This result highlights that, counterintuitively, two-stage processes need not necessarily be better than one-stage processes^18^ and that computational modeling, therefore, is a crucial step when designing such processes.

Further details regarding the case studies can be found in Section S3.

## Conclusion

Fed-batch processes with a growth-arrested production stage are an increasingly popular tool in modern bioprocessing. However, most researchers use custom spreadsheets or manual methods to design and iteratively improve their feeding strategies. Therefore, we developed FedBatchDesigner as an intuitive and accessible tool for optimizing such processes with minimal input requirements. By focusing on simplicity, our approach allows users to quickly explore the effect of different feed rates and switching times on the TRY metrics. Despite the simplifying assumptions underlying FedBatchDesigner, its results represent a robust first approximation (see Section S2 for details) of the productivity–titer trade-off and provide valuable insight without the need for coding skills, complex modeling, or extensive data sets.

The case studies presented here demonstrate how FedBatchDesigner can identify optimal feeding strategies and highlight potential improvements over existing experimental setups. The accompanying Jupyter Notebooks^19,22^ illustrate how the required parameters can be estimated from existing experimental data. With its interactive visualizations, open access availability, and ease of use, FedBatchDesigner enables researchers to make informed decisions about the optimal design of their fed-batch processes, ultimately accelerating bioprocess development.

## Supplementary Material





## Notes

Video demonstration (MP4)
Analytical solutions for 2SFB processes under multiple feeding strategies; sensitivity analysis of optimal switching time to inaccuracies in input parameters; additional case study on mevalonic acid production; links to source code and Jupyter Notebooks for case studies; supplementary Figures for case studies and sensitivity analysis; description of Video sections (PDF)

## List of acronyms

2SFB: two-stage fed-batch
ATP: adenosine triphosphate
TRY: titer, rate, and yield
