# Supplementary material for: Fedbatchdesigner: A User-Friendly Dashboard for Modeling and Optimizing Growth-Arrested Fed-Batch Processes
Source: ACS Synth Biol. 2025 Jul 21;14(8):3252–7. doi: 10.1021/acssynbio.5c00357 (PMC12362597; doi:10.1021/acssynbio.5c00357)
Supplement: Supplementary file 2 [file sb5c00357_si_002.pdf]

FedBatchDesigner: A user-friendly dashboard for modeling and optimizing growth-arrested fed-batch processes

Andrea C. Graf,[†,‡,‖] Julian Libiseller-Egger,[†,¶,‖] Mathias Gotsmy,[§] and Jürgen Zanghellini[*,†]

†*Department of Analytical Chemistry, University of Vienna, 1090 Vienna, Austria, EU*

‡*Austrian Centre of Industrial Biotechnology GmbH, 1190 Vienna, Austria, EU*

¶*Doctoral School in Chemistry, University of Vienna, 1090 Vienna, Austria, EU*

§*Department of Chemistry and Applied Biosciences, ETH Zurich, 8093 Zürich, Switzerland*

‖*Both authors contributed equally*

E-mail: juergen.zanghellini@univie.ac.at

Contents

1 Analytical solutions of the two-stage fed-batch (2SFB) S2

Bioreactor model . . . S2

Cell model . . . S4

Feasible feeding strategies . . . S5

Analytical solutions . . . S6

Exponential feeding . . . S7

Linear feeding . . . S8

Constant feeding . . . S9

2 Sensitivity of the predicted optimal switching time to inaccuracies in input parameters S11

3 Case studies **S12**
3.1 Additional case study: Production of mevalonic acid S12
3.2 Using `FedBatchDesigner` for anaerobic fermentation products S12
3.3 Parameter fitting for case studies S13

4 Video explanation **S13**

5 Supplementary Figures **S14**

6 Acronyms **S17**

References **S17**

1 Analytical solutions of the 2SFB

`FedBatchDesigner` is able to exhaustively evaluate the space of feed strategies quickly because of analytical expressions for the evolution of the biomass and product concentrations. The derivation of these expressions is detailed below.

Bioreactor model

We consider a fed-batch process with a growth-arrested production stage (Figure S1), subject to the assumptions outlined in the main manuscript. Feeding begins at the end of the batch phase, at $t = 0$.

During feeding, the time evolution of the concentrations of biomass x, product p, and substrate s in the bioreactor are governed by the following system of ordinary differential equations (ODEs)

$$\frac{\mathrm{d}xV}{\mathrm{d}t} = \mu x V, \qquad x(0) = x_0, \tag{1a}$$

$$\frac{\mathrm{d}pV}{\mathrm{d}t} = \pi x V, \qquad p(0) = 0, \tag{1b}$$

$$\frac{\mathrm{d}sV}{\mathrm{d}t} = -\sigma x V + f s_{\mathrm{f}}, \qquad s(0) = 0, \tag{1c}$$

$$\frac{\mathrm{d}V}{\mathrm{d}t} = f, \qquad V(0) = V_{\mathrm{batch}}. \tag{1d}$$

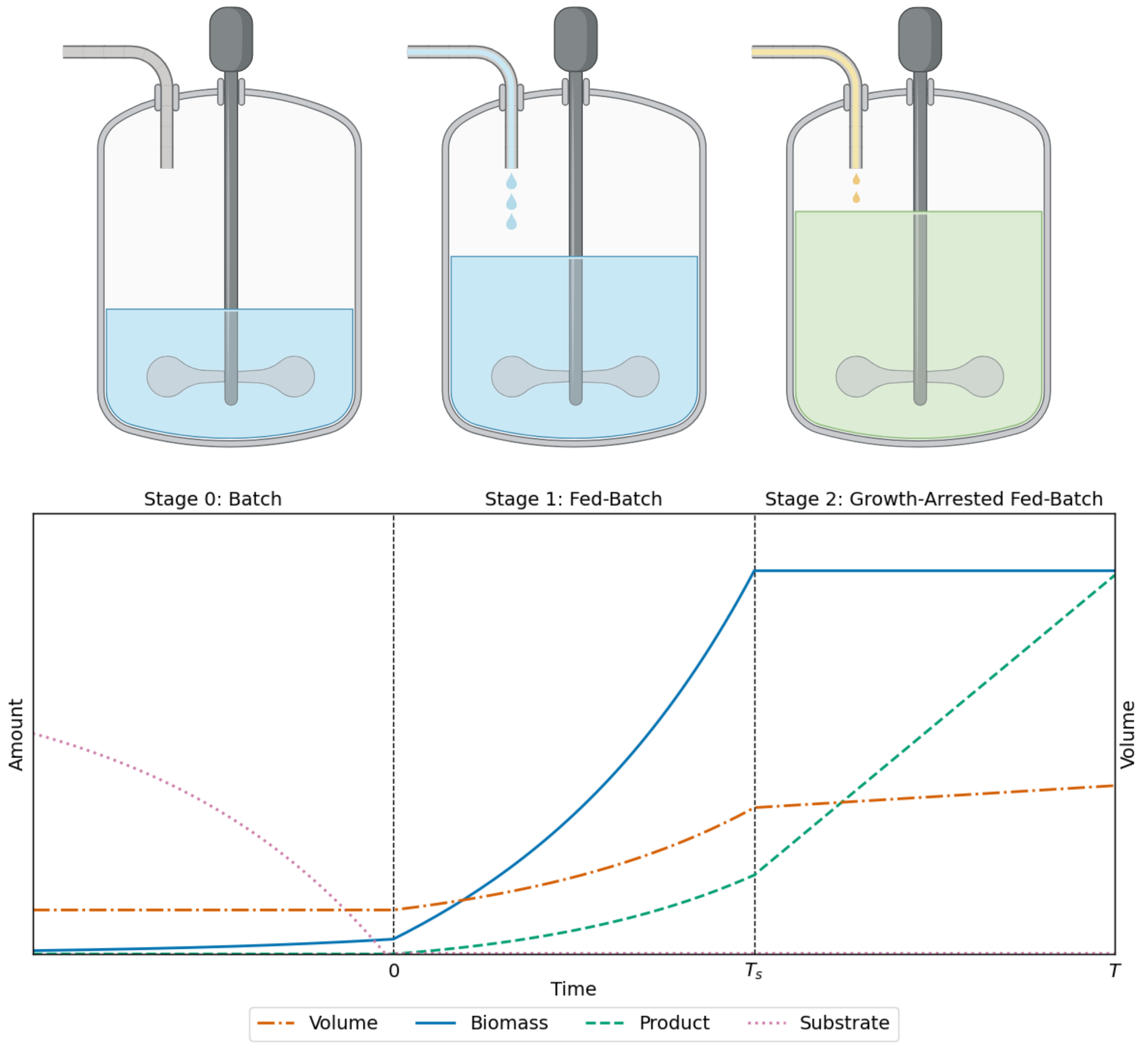

Figure S1: Schematic representation (top panels) and time evolution of key variables (bottom panels) of a fed-batch process with a growth arrested production phase. Created in BioRender.

Here, f denotes the feeding rate, V represents the current filling volume of the reactor, and V_{batch} the initial volume (i.e., the volume after the batch phase). x_0 is the biomass concentration at the end of the batch and s_{f} is the substrate concentration in the feed. The parameters μ, π, and σ describe the cells' specific growth rate, specific productivity, and specific substrate uptake rate, respectively.

In the following sections, we will derive functions that solve (1). Sometimes, these func-

tions (and their parameters) change depending on the stage, see Figure S1. When this happens, we use a single prime, «$'$», to indicate stage 1 and a double prime, «$''$», to indicate stage 2. For example, f' represents the feed rate in stage 1, while f'' represents the feed rate in stage 2. To avoid confusion with first and second derivatives, we will use Leibniz' notation ($\mathrm{d}f/\mathrm{d}t$) when expressing derivatives.

In accordance with our assumptions (see main manuscript), all substrate is consumed during the batch phase, while no product is produced. Thus, at the start of feeding, we have $p(0) = s(0) = 0$.

Since the substrate concentration remains zero throughout the fed-batch, it follows that $\mathrm{d}s/\mathrm{d}t = 0$. This means that the fed substrate is immediately consumed, which allows us to determine the substrate uptake rate

$$\sigma = \frac{f s_{\mathrm{f}}}{xV} \tag{2}$$

from (1c).

Cell model

We assume that the substrate is metabolized for three purposes: (i) to produce adenosine triphosphate (ATP) to provide energy for non-growth-associated maintenance, (ii) to synthesize the product, and (iii) to support cell growth. This gives the relation

$$\sigma = \frac{\mu}{Y_{\mathrm{x/s}}} + \frac{\pi}{Y_{\mathrm{p/s}}} + \frac{\rho}{Y_{\mathrm{atp/s}}}. \tag{3}$$

Here, $Y_{\mathrm{x/s}}$, $Y_{\mathrm{p/s}}$, and $Y_{\mathrm{atp/s}}$ represent the true biomass yield per substrate, the true product yield per substrate, and the true ATP yield per substrate, respectively, while ρ denotes the constant non-growth-associated ATP demand for maintenance.

Finally, we note that the specific product formation rate,

$$\pi = \pi_1 \mu + \pi_0, \tag{4}$$

is a linear function of μ, where π_1 and π_0 are the growth-dependent and growth-independent product formation coefficients.

Feasible feeding strategies

After combining (2), (3), and (4), we obtain

$$\mu = \alpha \left(\frac{s_\mathrm{f} Y_{\mathrm{x/s}}}{x} \frac{f}{V} - \beta \right) \tag{5a}$$

with

$$\alpha = \frac{1}{1 + \pi_1 \frac{Y_{\mathrm{x/s}}}{Y_{\mathrm{p/s}}}}, \tag{5b}$$

$$\beta = \pi_0 \frac{Y_{\mathrm{x/s}}}{Y_{\mathrm{p/s}}} + \rho \frac{Y_{\mathrm{x/s}}}{Y_{\mathrm{atp/s}}}. \tag{5c}$$

Here, $0 < \alpha \leq 1$ and $0 \leq \beta$ are scaling factors that account for the effect of growth-coupled production, π_1, and the combined effects of growth-independent production, π_0 and cellular maintenance, ρ, respectively. We note that growth is determined by the remaining substrate after accounting for maintenance and product formation (as noted in the list of assumptions in the main manuscript).

In (5a), we derived an expression for the growth rate, which remains valid as long as growth is controlled by substrate feeding. However, at time T_s, the process transitions to the production phase, where cellular growth halts due to external constraints, such as sulfate depletion (*1*, *2*) or genetically induced growth inhibition (*3*). Consequently, we must adjust the feed rate to ensure that the assumption of no substrate accumulation remains satisfied.

To determine the feed rate during the production stage, we set (5a) to zero, which yields:

$$f_0'' := f = \beta'' V(T_\mathrm{s}) \frac{x(T_\mathrm{s})}{s_\mathrm{f}'' Y_{\mathrm{x/s}}} = \text{const., for } T_\mathrm{s} \leq t \leq T. \tag{6}$$

Thus, during the production stage, the feed rate depends on the volume and biomass concentration at switching time T_s but remains constant, precisely matching the requirements for maintenance and growth-independent production, until the end of the fed-batch process at time T.

In summary, based on our assumptions (see main manuscript), we derived a feasible feeding strategy

$$f = f(t) = \begin{cases} f'(t) & \text{for } 0 \leq t \leq T_\mathrm{s}, \\ f_0''(T_\mathrm{s}) & \text{for } T_\mathrm{s} \leq t \leq T, \end{cases} \tag{7}$$

which is consistent with our bioreactor setup. Specifically, the feed rate $f'(t)$ can be chosen freely during the growth phase, whereas in the production phase, it remains constant at a fixed value f_0'', determined by the cell's requirements and switching time according to (6). This feeding strategy results in a specific growth rate

$$\mu = \mu(t) = \begin{cases} \alpha' \left(\frac{s_\mathrm{f}' Y_{\mathrm{x/s}}}{x(t)} \frac{f'(t)}{V(t)} - \beta' \right) & \text{for } 0 \leq t \leq T_\mathrm{s}, \\ 0 & \text{for } T_\mathrm{s} \leq t \leq T. \end{cases} \tag{8}$$

Analytical solutions

In the following, we consider exponential feeding during the growth stage

$$f' = f'(t; f_0', \mu_\mathrm{f}) = f_0' \exp(\mu_\mathrm{f} t), \quad 0 \leq t \leq T_\mathrm{s}. \tag{9}$$

Here, «′» denotes parameters referring to the growth stage, f_0' is the initial feeding rate, and μ_f represents the specific growth rate of the feed. For feasibility, we require $f_0' \geq f_0''$; see (6). We note that

for "small" μ_f, the expression in (9) can be approximated using a Taylor expansion:

$$f'(t; f_0', \mu_\mathrm{f}) = f_0' \left[1 + \mu_\mathrm{f} t + \frac{(\mu_\mathrm{f} t)^2}{2!} + \dots \right] \approx f_0'(1 + \mu_\mathrm{f} t) = f_0' + k_f t,$$

where $k_f = \mu_\mathrm{f} f'_0$ denotes the constant rate of increase in the feed. This corresponds to a *linearly increasing* feed profile.

In the limit $\mu_\mathrm{f} \to 0$, this further reduces to a *constant* feed profile:

$$\lim_{\mu_\mathrm{f} \to 0} f'(t; f'_0, \mu_\mathrm{f}) = f'_0 = \mathrm{const.}$$

Exponential feeding

With an exponential feed, the amount of biomass xV evolves as

$$xV = \begin{cases} \frac{\alpha' f'_0 s'_\mathrm{f} Y_\mathrm{x/s}}{\alpha'\beta' + \mu_\mathrm{f}} \mathrm{e}^{\mu_\mathrm{f} t} + \left[x_0 V_\mathrm{batch} - \frac{\alpha' f'_0 s'_\mathrm{f} Y_\mathrm{x/s}}{\alpha'\beta' + \mu_\mathrm{f}} \right] \mathrm{e}^{-\alpha'\beta' t} & \text{for } 0 \leq t \leq T_\mathrm{s}, \\ xV(T_\mathrm{s}) = \mathrm{const.} & \text{for } T_\mathrm{s} \leq t \leq T. \end{cases} \tag{10}$$

By selecting an initial feeding rate of

$$f'_0 = \left(\beta' + \frac{\mu_\mathrm{f}}{\alpha'} \right) \frac{x_0 V_\mathrm{batch}}{s'_\mathrm{f} Y_\mathrm{x/s}}, \tag{11}$$

the term in square brackets above vanishes, ensuring that the cells immediately grow exponentially without any transient adaptation phase.

For the total biomass, product, and volume, we get

$$xV = x_0 V_{\mathrm{batch}} \times \begin{cases} \exp(\mu_{\mathrm{f}} t) & \text{for } 0 \le t \le T_{\mathrm{s}}, \\ \exp(\mu_{\mathrm{f}} T_{\mathrm{s}}) & \text{for } T_{\mathrm{s}} \le t \le T, \end{cases} \tag{12a}$$

$$pV = \begin{cases} \left(\pi_1' + \frac{\pi_0'}{\mu_{\mathrm{f}}}\right) \cdot x_0 V_{\mathrm{batch}} \cdot [\exp(\mu_{\mathrm{f}} t) - 1] & \text{for } 0 \le t \le T_{\mathrm{s}}, \\ \pi_0'' \cdot (t - T_{\mathrm{s}}) \cdot xV(T_{\mathrm{s}}) + pV(T_{\mathrm{s}}) & \text{for } T_{\mathrm{s}} \le t \le T, \end{cases} \tag{12b}$$

$$sV = 0 \quad \text{for } 0 \le t \le T, \tag{12c}$$

$$V = \begin{cases} \frac{f_0'}{\mu_{\mathrm{f}}} \cdot [\exp(\mu_{\mathrm{f}} t) - 1] + V_{\mathrm{batch}} & \text{for } 0 \le t \le T_{\mathrm{s}}, \\ f_0'' \cdot (t - T_{\mathrm{s}}) + V(T_{\mathrm{s}}) & \text{for } T_{\mathrm{s}} \le t \le T, \end{cases} \tag{12d}$$

where f_0' and f_0'' are given by (11), and (6), respectively. For simplicity, we use the shorthand notation $xV = xV(t) = x(t) \cdot V(t)$ to represent the total amount of biomass at time t. The same shorthand applies to pV.

$\frac{f_0'}{\mu_{\mathrm{f}}}$ corresponds to the "substrate start volume" (i.e., the volume usually used in bioreactor control software to set up exponential feed strategies) and calculating it according to (11) can be thought of as choosing a substrate start volume that takes the substrate required for non-growth processes into account. In other words, the initial feed rate according to (11) ensures that biomass grows at the desired rate despite maintenance and product formation.

Linear feeding

To obtain the biomass evolution during a linearly increasing feed profile, we approximate the exponential feeding case given in (10) by considering the regime of small μ_{f}. Specifically, we assume $0 < \mu_{\mathrm{f}} t \ll 1$ and $0 < \mu_{\mathrm{f}} \ll \alpha' \beta'$, which allows us to perform a first-order Taylor

expansion in μ_f. Using $k_f' = \mu_\mathrm{f} f_0'$, this yields:

$$xV = \begin{cases} \frac{s_\mathrm{f}' Y_\mathrm{x/s}}{\beta'} \left[f_0' - \frac{k_f'}{\alpha'\beta'} + k_f' t + \left(\frac{\beta' x_0 V_\mathrm{batch}}{s_\mathrm{f}' Y_\mathrm{x/s}} + \frac{k_f'}{\alpha'\beta'} - f_0' \right) \mathrm{e}^{-\alpha'\beta' t} \right] & \text{for } 0 \le t \le T_\mathrm{s}, \\ xV(T_\mathrm{s}) = \text{const.} & \text{for } T_\mathrm{s} \le t \le T. \end{cases} \tag{13}$$

We again select the initial feeding rate

$$f_0' = \frac{\beta' x_0 V_\mathrm{batch}}{s_\mathrm{f}' Y_\mathrm{x/s}} + \frac{k_f'}{\alpha'\beta'} \tag{14}$$

such that the transient behavior, determined by the expression in parentheses, vanishes.

Thus, tor the total biomass, product, and volume, we get

$$xV = x_0 V_\mathrm{batch} + \frac{s_\mathrm{f}' Y_\mathrm{x/s}}{\beta'} \times \begin{cases} k_f' t & \text{for } 0 \le t \le T_\mathrm{s}, \\ k_f' T_\mathrm{s} & \text{for } T_\mathrm{s} \le t \le T, \end{cases} \tag{15a}$$

$$pV = \begin{cases} x_0 V_\mathrm{batch} \pi_0' t + \frac{1}{2\beta} k_f' s_\mathrm{f}' Y_\mathrm{x/s} t \left(\pi_0' t + 2\pi_1' \right) & \text{for } 0 \le t \le T_\mathrm{s}, \\ \pi_0'' \cdot (t - T_\mathrm{s}) \cdot xV(T_\mathrm{s}) + pV(T_\mathrm{s}) & \text{for } T_\mathrm{s} \le t \le T, \end{cases} \tag{15b}$$

$$sV = 0 \quad \text{for } 0 \le t \le T, \tag{15c}$$

$$V = \begin{cases} f_0' t + k_f' \frac{t^2}{2} + V_\mathrm{batch} & \text{for } 0 \le t \le T_\mathrm{s}, \\ f_0'' \cdot (t - T_\mathrm{s}) + V(T_\mathrm{s}) & \text{for } T_\mathrm{s} \le t \le T. \end{cases} \tag{15d}$$

Constant feeding

We note that in the limit $\mu_\mathrm{f} \to 0$, the exponential feed reduces to a constant feeding profile. However, since the initial feeding rate f_0' is chosen according to (11), we have $f_0' \to f_0''$. As discussed above, see (6), this feed rate only meets the demands for maintenance and growth-independent production. Consequently, under these conditions, the system directly enters the production stage without first increasing biomass. For general constant feed, the

solutions to (1) read

$$xV = \begin{cases} \frac{f_0' s_\mathrm{f}' Y_\mathrm{x/s}}{\beta'} + \left[x_0 V_\mathrm{batch} - \frac{f_0' s_\mathrm{f}' Y_\mathrm{x/s}}{\beta'}\right] \mathrm{e}^{-\alpha'\beta' t} & \text{for } 0 \le t \le T_\mathrm{s}, \\ xV(T_\mathrm{s}) = \text{const.} & \text{for } T_\mathrm{s} \le t \le T, \end{cases} \tag{16a}$$

$$pV = \begin{cases} \frac{\pi_0'}{\beta'} f_0' s_\mathrm{f} Y_\mathrm{x/s} t + \left(\frac{\pi_0'}{\alpha'\beta'} - \pi_1'\right)\left(x_0 V_\mathrm{batch} - \frac{\pi_0'}{\beta'} f_0' s_\mathrm{f}' Y_\mathrm{x/s}\right)\left[1 - \mathrm{e}^{-\alpha'\beta' t}\right] & \text{for } 0 \le t \le T_\mathrm{s}, \\ \pi_0'' \cdot (t - T_\mathrm{s}) \cdot xV(T_\mathrm{s}) + pV(T_\mathrm{s}) & \text{for } T_\mathrm{s} \le t \le T, \end{cases} \tag{16b}$$

$$sV = 0 \quad \text{for } 0 \le t \le T, \tag{16c}$$

$$V = \begin{cases} f_0' t + V_\mathrm{batch} & \text{for } 0 \le t \le T_\mathrm{s}, \\ f_0'' \cdot (t - T_\mathrm{s}) + V(T_\mathrm{s}) & \text{for } T_\mathrm{s} \le t \le T. \end{cases} \tag{16d}$$

With the solutions to (1) at hand, describing the time evolution of biomass, product, and substrate, we aim to maximize the average volumetric productivity

$$\max_{T_\mathrm{s}} \left[\frac{pV(T)}{T \cdot V(T)}\right] = \frac{1}{V_\mathrm{max}} \max_{T_\mathrm{s}} \left[\frac{pV(T)}{T}\right]. \tag{17}$$

Since the bioreactor's capacity is constrained, we will fix the final volume $V_\mathrm{max} = V(T)$. It is therefore convenient to express the duration of the fed-batch process, T, as a function of V_max according to

$$T = \frac{1}{f_0''}\left[V_\mathrm{max} - V(T_\mathrm{s})\right] + T_\mathrm{s}. \tag{18}$$

By substituting this expression into (17), differentiating the resulting equation with respect to T_s, and setting the derivative to zero, we obtain a transcendental equation for the optimal switching time, T_s. In general, a solution to this equation is only available through numerical methods.

2 Sensitivity of the predicted optimal switching time to inaccuracies in input parameters

When using `FedBatchDesigner`, the physiological parameters ρ, π_0, and π_1 as well as the per-substrate yields $Y_{x/s}$, $Y_{p/s}$, and $Y_{atp/s}$ are either estimated from experimental data or extracted from the literature. Therefore, due to measurement error or differences in experimental setup, the selected numbers can deviate from the actual values. Figure S4 illustrates these effects for the case study of valine production in *E. coli*. It shows how the main results of `FedBatchDesigner`, the switching time from growth to production stage and the highest-achievable space-time yield, are affected when random noise (with a relative standard deviation of 10%) is added to these parameters. The influence on the outputs was moderate, with effects smaller than 10% in the majority of cases. The calculation of the switching time was the most robust when considering exponential feed, whereas with constant feed the effect was more pronounced and thus accurate parameter estimation is more important. However, the overall small magnitude of deviation in the results indicates that, even when the estimates of the parameters required by `FedBatchDesigner` are not very precise, it delivers a good first approximation of the productivity–titer trade-off, which is useful to inform subsequent experiments as it can be obtained from very little data.

3 Case studies

3.1 Additional case study: Production of mevalonic acid

Masuda et al. (*1*) identified mevalonate synthesis in an engineered *E. coli* strain as a promising candidate for growth-decoupled production, where sulfur starvation prevents growth and enhances product accumulation. Based on the data they gained in shake flask cultivations, we estimated the specific productivities required for using `FedBatchDesigner` (see the Jupyter Notebook at (*4*) for details). We applied these parameters to design a 5 L 2SFB process with exponential feed, achieving a titer of $109\,g\,L^{-1}$ at a yield of $0.455\,g\,g^{-1}$ and an average volumetric productivity of $2.98\,g\,L^{-1}\,h^{-1}$ (assuming a 4-hour batch phase). To the best of our knowledge, this is comparable to the highest mevalonate titers reported in the literature, but at considerably greater average volumetric productivity ($111\,g\,L^{-1}$ and $1.5\,g\,L^{-1}\,h^{-1}$, respectively) (*5*, *6*).

3.2 Using `FedBatchDesigner` for anaerobic fermentation products

In anaerobic fermentative production processes, such as ethanol production by *S. cerevisiae* (case study 2), ATP generation is inherently coupled to product formation. In these systems, substrate consumption for cell maintenance becomes negligible, as the energy required is typically met by the same metabolic pathways used for product synthesis. In other words, in the aerobic case, substrate is used to either cover the cell's maintenance costs or for product formation (or for growth). In the anaerobic case, on the other hand, the ATP required for maintenance is provided by substrate-level phosphorylation concomitant with product (e.g. ethanol) formation. Therefore, no additional substrate is consumed to satisfy the maintenance requirement.

The `FedBatchDesigner` user interface has a checkbox for this case which, when ticked, sets the maintenance rate parameter to zero.

3.3 Parameter fitting for case studies

The parameters required for the case studies described in the main text are estimated in Jupyter Notebooks at

- `https://github.com/julibeg/FedBatchDesigner/blob/main/case-studies/valine/fit-parameters.ipynb`
- `https://github.com/julibeg/FedBatchDesigner/blob/main/case-studies/ethanol-atp-wasting/fit-parameters.ipynb`
- `https://github.com/julibeg/FedBatchDesigner/blob/main/case-studies/mevalonate/fit-parameters.ipynb`

4 Video explanation

The Supplementary Files include a video demonstrating the use of `FedBatchDesigner`, focusing specifically on the valine case study. It covers the following topics:

- 00:00 – Introduction to `FedBatchDesigner`
- 00:25 – `FedBatchDesigner` output
- 01:18 – L-Valine case study
- 02:07 – Jupyter notebook for data fitting
- 03:23 – `FedBatchDesigner` input mask
- 03:41 – `FedBatchDesigner` constant feed recreation of L-Valine case study
- 04:23 – Exponential process design with `FedBatchDesigner`

5 Supplementary Figures

(a)

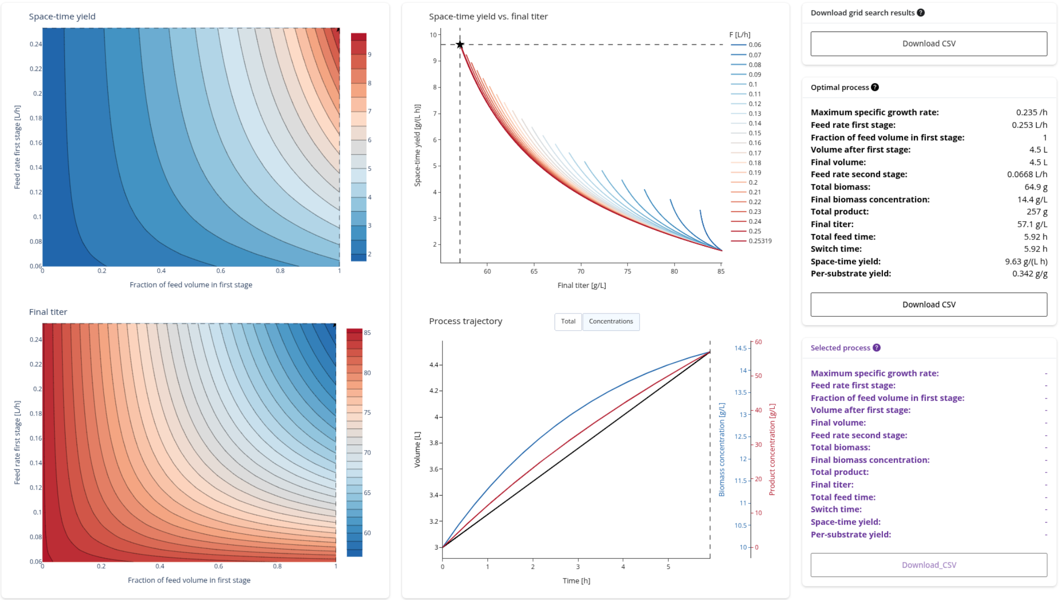

(b)

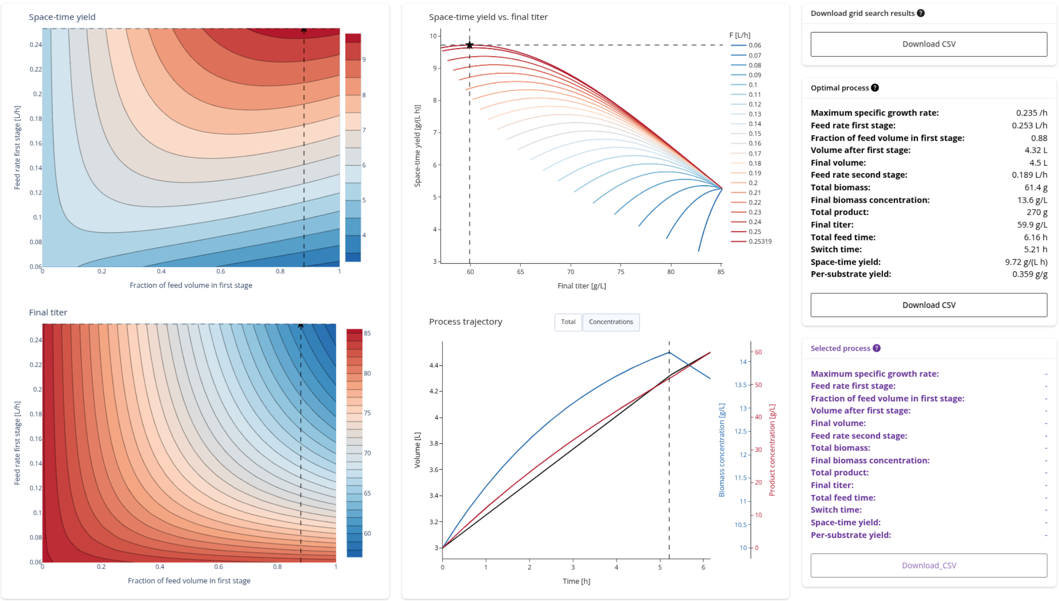

Figure S2: Ethanol production with ATP wasting: with the parameters estimated from the data in (*7*) (see notebook in (*8*) for details on the fitting process), volumetric productivity is greatest for the one-stage fed-batch (a). The ethanol formation rate in the production phase would need to increase threefold (while keeping all other parameters constant) in order to achieve higher volumetric productivity with a two-stage feed strategy (b).

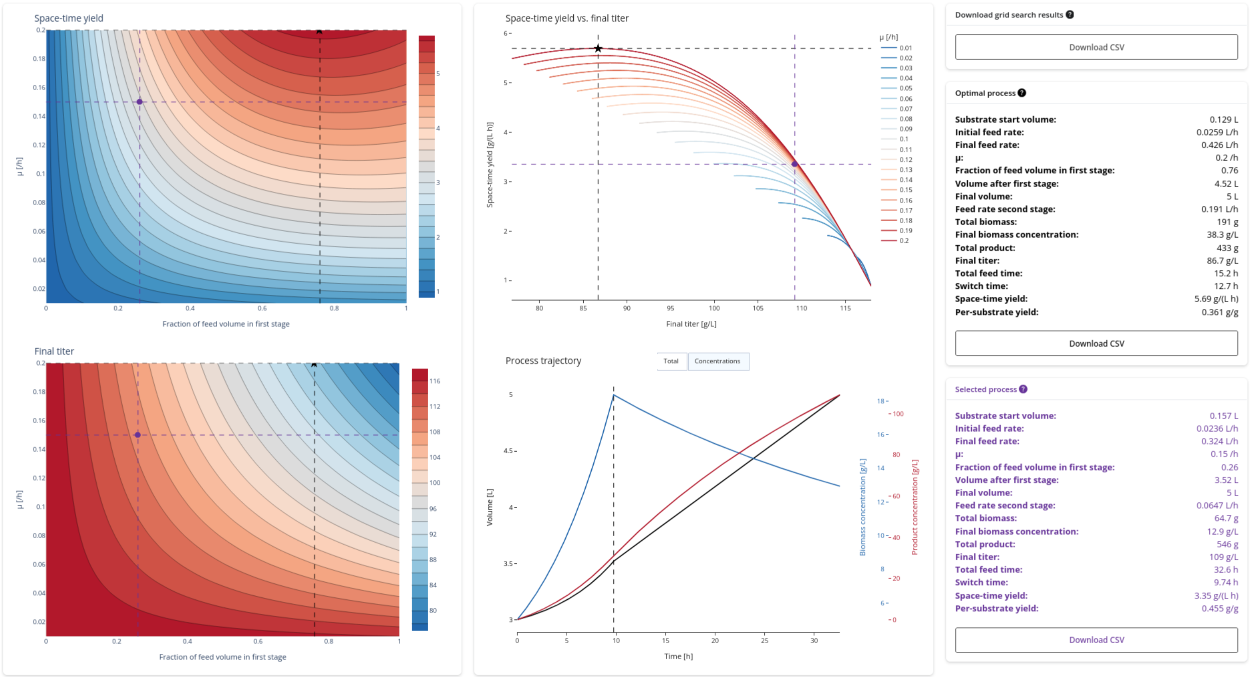

Figure S3: `FedBatchDesigner`'s output panels for designing a sulfur-limited 2SFB process of mevalonate production in engineered *E. coli*. The tool predicts that a titer of $109\,\mathrm{g\,L^{-1}}$, with average volumetric productivity of $2.98\,\mathrm{g\,L^{-1}\,h^{-1}}$ (assuming a batch duration of 4 hours) and a yield of $0.455\,\mathrm{g\,g^{-1}}$ can be reached in a 5 L 2SFB with exponential feed.

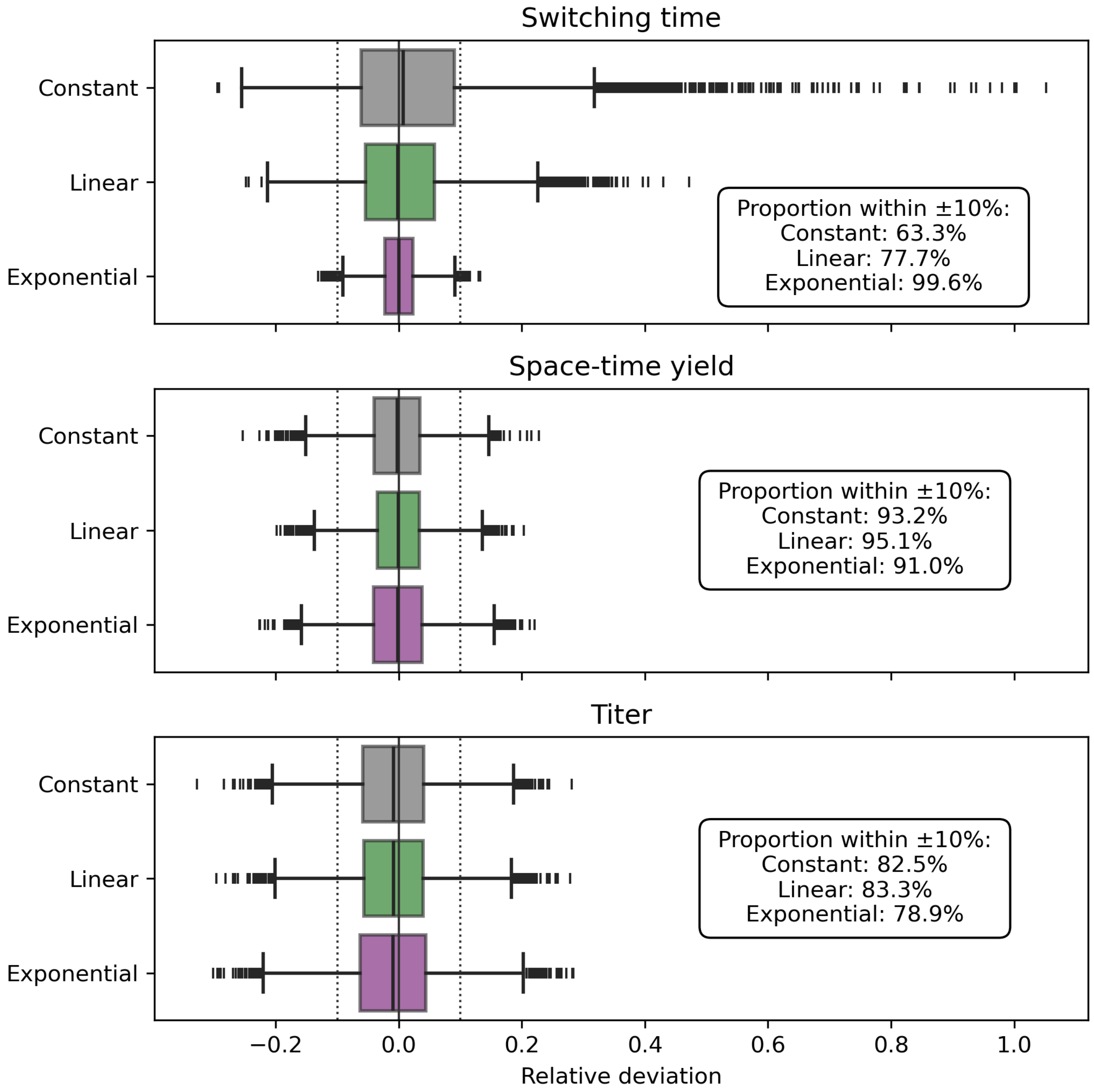

Figure S4: Based on the valine production case study, this illustrates the relative change in optimal switching time as well as the corresponding titer and space-time yield when adding Gaussian noise to the values of the physiological parameters required by `FedBatchDesigner` (the maintenance requirement ρ, specific product formation rates π_0 and π_1 as well as the per-substrate yields $Y_{x/s}$, $Y_{p/s}$, and $Y_{atp/s}$). The box plots show the results of $N = 10,000$ draws from normal distributions (i.e., for each parameter a new value was drawn with the original value as mean and a relative standard deviation of 10%; the optimal process was determined for 10,000 such randomized parameter sets).

6 Acronyms

2SFB two-stage fed-batch

ATP adenosine triphosphate

TRY titer, rate, and yield

References

1. Masuda, A., Toya, Y., and Shimizu, H. (2017) Metabolic Impact of Nutrient Starvation in Mevalonate-Producing Escherichia Coli. *Bioresource Technology 245*, 1634–1640.

2. Gotsmy, M., Strobl, F., Weiß, F., Gruber, P., Kraus, B., Mairhofer, J., and Zanghellini, J. (2023) Sulfate Limitation Increases Specific Plasmid DNA Yield and Productivity in E. Coli Fed-Batch Processes. *Microbial Cell Factories 22*, 242.

3. Stargardt, P., Feuchtenhofer, L., Cserjan-Puschmann, M., Striedner, G., and Mairhofer, J. (2020) Bacteriophage Inspired Growth-Decoupled Recombinant Protein Production in Escherichia Coli. *ACS Synthetic Biology 9*, 1336–1348.

4. Case Study Mevalonate Jupyter Notebook. `https://github.com/julibeg/FedBatchDesigner/blob/main/case-studies/mevalonate/fit-parameters.ipynb`.

5. Zhu, Y., Li, Y., Xu, Y., Zhang, J., Ma, L., Qi, Q., and Wang, Q. (2021) Development of Bifunctional Biosensors for Sensing and Dynamic Control of Glycolysis Flux in Metabolic Engineering. *Metabolic Engineering 68*, 142–151.

6. Wang, C.-H., Hou, J., Deng, H.-K., and Wang, L.-J. (2023) Microbial Production of Mevalonate. *Journal of Biotechnology 370*, 1–11.

7. Zahoor, A., Messerschmidt, K., Boecker, S., and Klamt, S. (2020) ATPase-based Implementation of Enforced ATP Wasting in Saccharomyces Cerevisiae for Improved Ethanol Production. *Biotechnology for Biofuels 13*, 185.

8. Case Study Ethanol ATP Wasting Jupyter Notebook. `https://github.com/julibeg/FedBatchDesigner/blob/main/case-studies/ethanol-atp-wasting/fit-parameters.ipynb`.
